# State Variation in Medicare Reimbursement for the Top 100 Cardiothoracic Surgery Procedures, 2013-2022

**DOI:** 10.7759/cureus.70275

**Published:** 2024-09-26

**Authors:** Jack G Allen, Carson Bateman, Alexander Dorius, Alan Pang, John Griswold, William Springer

**Affiliations:** 1 Department of Surgery, Texas Tech University Health Sciences Center, Lubbock, USA; 2 Department of Surgery, University of Utah School of Medicine, Salt Lake City, USA; 3 Department of Cardiothoracic Surgery, Texas Tech University Health Sciences Center, Lubbock, USA

**Keywords:** cardiothoracic surgery, economics, healthcare policy, medicare, reimbursement

## Abstract

Objective

We analyzed changes in reimbursement rates for cardiothoracic surgery procedures from 2013 to 2022 to identify interstate variance and compare changes in reimbursement between procedural groups.

Methods

The Center for Medicare and Medicaid Services database was analyzed to find the 100 highest-grossing cardiothoracic surgery CPT codes from 2013 to 2022. Medicare Administrative Contractor codes were utilized to identify reimbursement for each state. The payments were adjusted for inflation utilizing the consumer price index. Procedures were weighted according to revenue, and an average yearly, inflation-adjusted percent change in reimbursement was generated for each U.S. state. Procedural groups were compared using single-factor ANOVA and post-hoc tests.

Results

Since 2013, the inflation-adjusted Medicare reimbursement for the top cardiothoracic surgery procedures for all U.S. states and territories decreased by a yearly average of 2.67%. Puerto Rico (-0.33%), Louisiana (-1.84%), and Alabama (-1.85%) showed the smallest change. Illinois (-3.62%), Kansas (-3.40%), and Wyoming (-3.22%) had the greatest decrease in reimbursement throughout the observed period. Pacemaker and defibrillator (mean: -20.85), valvular (-22.07%), and coronary artery bypass graft (CABG) surgery (-21.99%) procedural groups demonstrated significant differences between valvular (p=0.01) or CABG (p=0.02) and pacemaker and defibrillator.

Conclusions

Our study confirms that reimbursement patterns vary by geographical area. Second, geographic variation suggests an incentive for physicians to practice in states with higher Medicare reimbursement. Certain procedural groups have been affected more than others. New lobbying strategies may be needed to mitigate diminishing reimbursement so that quality of care is not impacted for Medicare beneficiaries in low reimbursement states.

## Introduction

Medicare is currently the largest insurer in the United States and was established to provide health insurance for the elderly with qualifying health conditions. The rapid expansion of Medicare in recent decades is largely attributed to the aging population in the United States. With an increasing proportion of patients relying on Medicare, it is essential to critically assess reimbursement rates and their implications for both healthcare providers and patient care [1,2].

Physician insurance reimbursement in cardiothoracic surgery is based on the procedure performed and codified into a Current Procedural Terminology (CPT) code. The CPT code allows the physician to bill the insurance company for the provided services. CPT codes are assigned a sum of relative value units (RVU) that determines the payout received from the insurance company for the procedure performed. Medicare factors total RVUs by summing work RVUs (wRVUs), practice expense RVUs (pRVUs), and practice liability expense or malpractice RVUs (mRVUs). Generally, the majority of insurance payments are based on these Medicare reimbursement rates, necessitating this study [3,4].

Additionally, Medicare RVUs are adjusted by geography using the geographic practice cost index (GPCI), which varies by state or territory within the United States [1,3]. GPCI attempts to make physician pay “equalized” across differing geographic regions by accounting for differences in labor markets, real estate, malpractice insurance, purchased services, supplies, and equipment. Additionally, some GPCI adjustments are chosen based on the cost of living and the desirability of a place to work and live. Therefore, areas attracting more physicians and having a lower cost of living will negatively impact GPCI adjustments, thereby decreasing Medicare payment in the respective region. According to the report from the committee on Geographic Adjustment Factors in Medicare Payment, these GPCI adjustments may not well target physicians in medically underserved or rural areas. Despite GPCI adjustments being the core influencer on geographic differences, the agranular representation of these changes will be represented using total Medicare reimbursement for a given procedure [5].

Decreasing Medicare reimbursement rates have been observed across multiple surgical and medical specialties, including orthopedic surgery, cardiothoracic surgery, neurosurgery, and dermatology [2,6-8]. These reductions stem from several factors, including federal budget cuts, an emphasis on increasing reimbursement for primary care, inflation, and the fact that Medicare operates under a balanced budget framework. Medicare reimbursement is determined by the Resource-Based Relative Value Scale (RBRVS), which assigns a value to medical procedures based on physician work, practice expense, and malpractice costs, with adjustments for geographic regions and inflation. Though some studies have examined geographic variance in other specialties, no studies have examined the impact of geography on Medicare reimbursement rates in cardiothoracic surgery or the impacts of possible geographic variance [9-11].

This study sought to identify geographic differences in Medicare reimbursement changes for cardiothoracic surgery between 2013 and 2022 and compare national reimbursement changes for significant cardiothoracic surgery procedural groups. We hypothesize that total reimbursement will decrease for cardiothoracic surgery procedures and that there will be geographic variance in the level to which reimbursement decreases. We also hypothesize that geographic changes in cardiothoracic surgeon distribution will correlate with these geographic differences in Medicare reimbursement. Finally, we hypothesize that there will be significant mean differences between procedural groups.

## Materials and methods

This study utilized open-access data found through the Center for Medicare and Medicaid Services Physician Fee Schedule (CMS-PFS) and, therefore, did not require IRB approval. Cardiothoracic surgery-specific CPT codes were identified from the coding office at Texas Tech University Health Sciences Center Department of Cardiothoracic Surgery and confirmed via the American Medical Association [12]. The CPT code range utilized in this analysis was 33016-37799. CPT codes that did not exist for the entirety of the studied period were excluded from the analysis.

Reimbursement data from 2013 to 2022 were accessed via the CMS-PFS look-up tool. We then identified the top 100 cardiothoracic surgery CPT codes based on the highest gross revenue and used these codes in our analysis. Using Mathematica, we processed the data and identified the total percent change in Medicare reimbursement for each year in the data set and the total change across the study period. The data was tabulated according to Medicare Administrative Contractor localities to identify changes in reimbursement for each state and territory.

A weighted average over the 2013 to 2022 period was calculated by pulling each procedure’s CPT code’s facility price from the CMS-PFS for each respective year. The procedural and payment data was then aggregated and the total revenue for each procedure was factored to weight each procedure:

$\text{Procedure Weight} = \frac{(\text{Annual Procedural Volume} \times \text{Procedural Allowed Payment})}{\text{Total Annual Revenue for all Procedures}}$

The weighting of each procedure as a portion of total annual revenue was thereafter used to weigh the relative change in Medicare reimbursement from 2013 to 2022. The 2014 facility price for each procedure was then updated for inflation in 2023 dollars using the Consumer Price Index from the Bureau of Labor Statistics. The weighted average change in Medicare rates from 2013 to 2022 was calculated for each subspecialty using the following formula:

$\text{Weighted Average Change} = \sum \left( \text{Procedure Weight} \times \left[ \left( \frac{\text{Facility Price}_{2022}}{\text{Facility Price}_{2013}} - 1 \right) \times 100\% \right] \right)$

This weighted average change in Medicare rates represents the theoretical financial impact by adjusting for the revenue of a procedure using volume and facility price.

Without adjusting for inflation, changes in Medicare reimbursement would be largely indiscernible since we retrospectively studied previous reimbursement rates. Reimbursement rates for each CPT code were adjusted for inflation using the Consumer Price Index provided by the U.S. Department of Labor. The geographic analysis was performed by the authors using Mathematica.

Using the Centers for Medicare and Medicaid Physician and Other Practitioners, by Provider dataset, years 2013 and 2021 were filtered by “Cardiothoracic Surgery” to count all surgeons by state. The dataset reported no cardiothoracic surgeons in Guam or the Virgin Islands, and these territories were omitted from the analysis. In sum, 52 states and territories were analyzed. The total percent change in cardiothoracic surgeons by state or territory from 2013 and 2021 was calculated. Normality of the total percent change by Medicare reimbursement and total percent change by cardiothoracic surgeons by state were assessed using the Shapiro-Wilk test. The total percent change by Medicare reimbursement for each state was not normally distributed (p<0.001). The linear relationship between the total percent change by Medicare reimbursement and total percent change by cardiothoracic surgeons by state were, therefore, analyzed using the non-parametric, Kendall’s Tau test.

The cardiothoracic surgery CPT codes that were analyzed were grouped by categories: coronary artery bypass graft surgery (CABG), valvular surgery, pericardial surgery, pacemaker and defibrillator surgery, and great vessel surgery. Only categories that comprised at least ten percent of the total gross revenue of all analyzed codes were analyzed. CABG, valvular surgery, and pacemaker and defibrillator surgery fit this criterion.

The national total percent change was factored for each procedure, and means were compared using IBM SPSS Statistics. An α of 0.05 was used to calculate statistical significance. Variance was assessed with the Levene test, which was significant (p<0.001). A single-factor analysis of variance (ANOVA) revealed a significant difference between groups (p=0.022). The Games-Howell post hoc test was chosen to determine the significance of mean differences between procedural groups because of unequal variances between groups.

## Results

**Figure 1:**
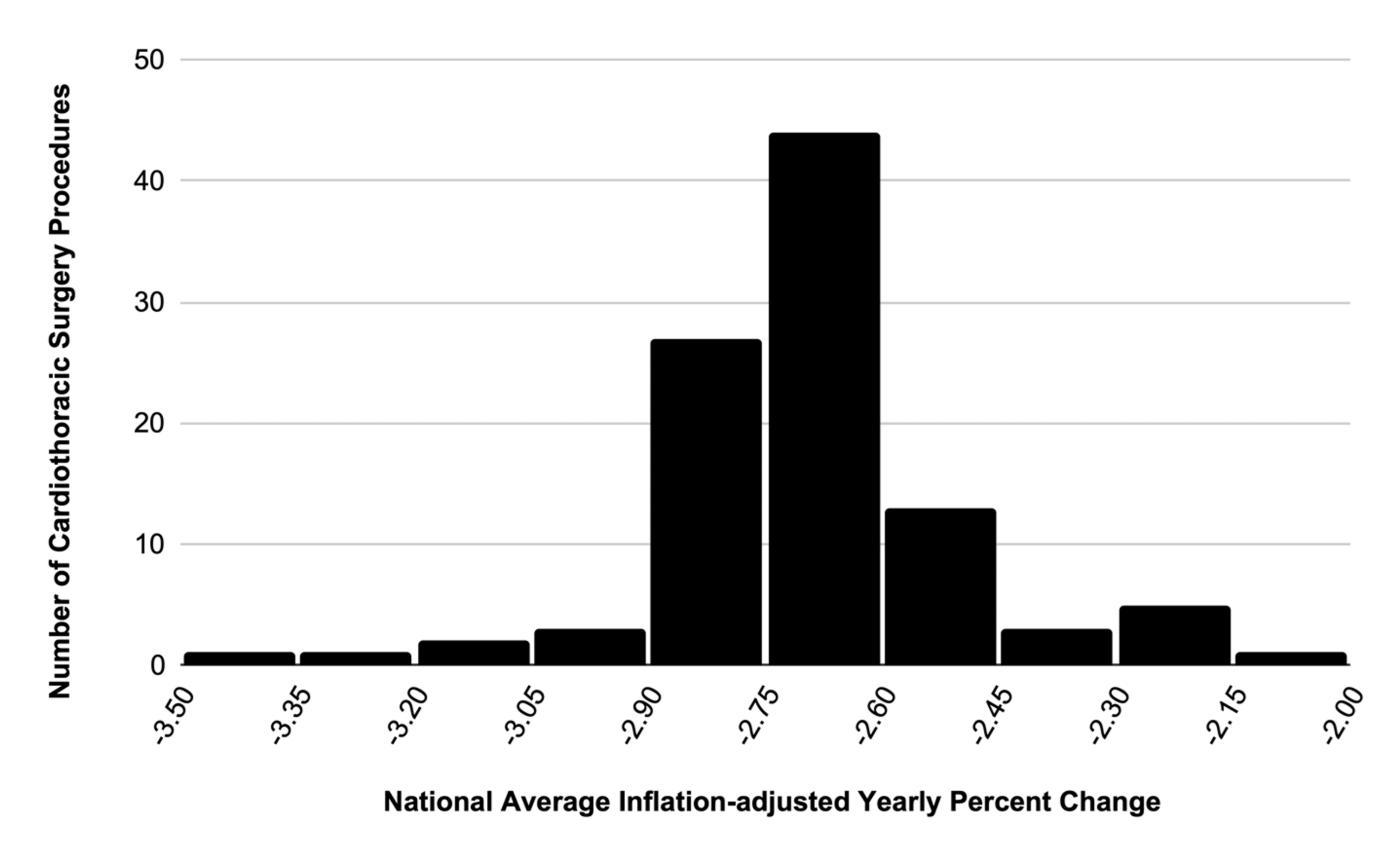
This histogram illustrates the distribution of inflation-adjusted yearly percent change for each of the cardiothoracic surgery procedures analyzed. Figure Credits: Jack Allen and Carson Bateman

Overall, the average change in inflation-adjusted Medicare reimbursement for the 100 cardiothoracic surgery procedures we examined for the 10-year period was -2.67% per year or a total national change of -21.35%. Figure 1 shows the distribution of the national average yearly percent change in Medicare reimbursement for each of the CPT codes examined. The distribution was largely regular.

The range for the national average inflation-adjusted yearly percent change was -3.46% to -2.15% with a median and mean of -2.67% and -2.73%, respectively. The highest-grossing procedures included arterial CABG (33533), aortic valve replacement (33405), and insertion of new or replacement of permanent pacemaker with transvenous electrodes (33208) were 28.05%, 12.94%, and 8.21% of the analyzed procedures and decreased by -2.66%, -2.67%, and -2.50%, respectively.

**Figure 2:**
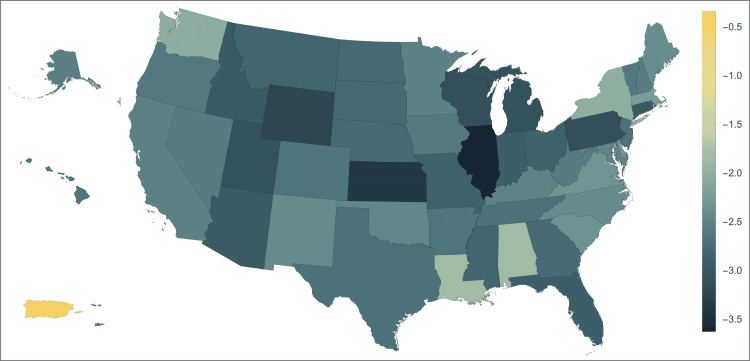
This map exhibits the average inflation-adjusted yearly percent change by state or territory. The shade of each state or territory corresponds to the percent change labeled in the key. Figure Credits: Jack Allen and Carson Bateman

Figure 2, Table 1, and Table 2 demonstrate geographic variance in changes in Medicare reimbursement rates. Physicians in Illinois (-3.62%), Kansas (-3.40%), and Wyoming (-3.22%) experienced steep declines in inflation-corrected reimbursement over the study period. This may lead to diminished wages for cardiothoracic surgeons in these states. This also may put a strain on hospital systems. However, surgeons in Louisiana (-1.84%), Alabama (-1.85%), and New York (-1.99%) experienced a lower decline in reimbursement and, therefore ,a probable lessened impact on wages. Understanding these trends can help cardiothoracic surgeons advocate for reimbursement that will keep pace with inflation. The inflation adjusted change in Medicare reimbursement for all states is included in the supplemental table.

**Table 1:** This table illustrates the total percent change and yearly average change in inflation-adjusted Medicare reimbursement for the least affected states across the 10-year period.

Least Affected States	Total % Change	Yearly % Change
Puerto Rico	-2.95	-0.33
Louisiana	-15.41	-1.84
Alabama	-15.48	-1.85
New York	-16.58	-1.99
Washington	-16.94	-2.04

**Table 2:** This table exhibits the total percent change and yearly average change in inflation-adjusted Medicare reimbursement for the most affected states across the 10-year period.

Most Affected States	Total % Change	Yearly % Change
Wisconsin	-24.77	-3.11
Pennsylvania	-24.85	-3.12
Wyoming	-25.58	-3.23
Kansas	-26.77	-3.40
Illinois	-28.27	-3.62

The correlation analysis revealed a Kendall’s Tau of τ=-0.09 between the total percent change by Medicare reimbursement and total percent change by cardiothoracic surgeons for the 52 states and territories. The correlation was not statistically significant (p=0.35), indicating no linear relationship between the total percent change by Medicare reimbursement and total percent change by cardiothoracic surgeons. The null hypothesis was accepted that geographic changes in cardiothoracic surgeon distribution does not correlate with geographic differences in Medicare reimbursement.

**Figure 3:**
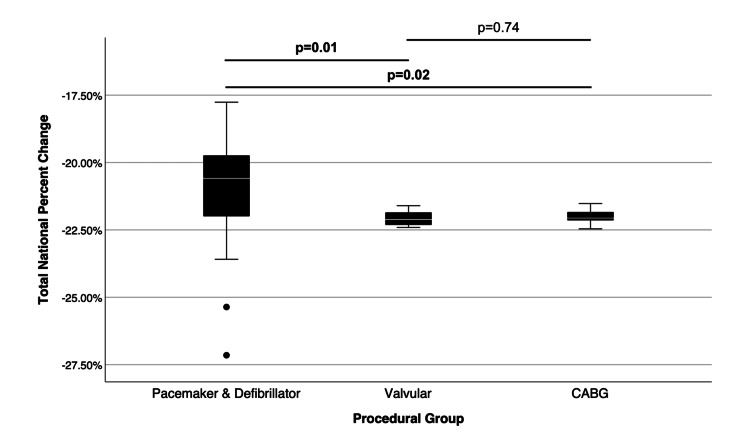
This box plot of pacemaker and defibrillator (n=28), valvular (12), and CABG (15) procedural groups for cardiothoracic surgery illustrates the total national percent change. Procedural group means were compared using the Games-Howell post hoc test and statistical differences were represented as bars and p-values between procedural groups. CABG, coronary artery bypass graft Figure Credits: Jack Allen and Alexander Dorius

**Figure 4:**
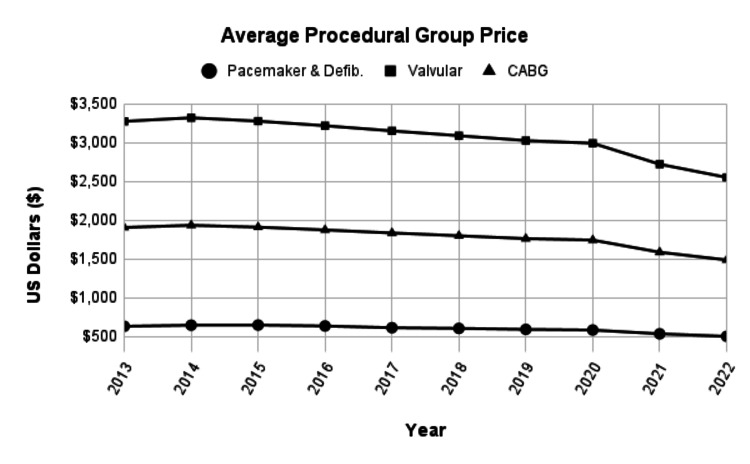
The line graphs identify changes in inflation-adjusted, average Medicare reimbursement rates for valvular procedures (CPT codes 33361-33478), pacemaker and defibrillator procedures (CPT codes 33202-33249), and CABG procedures (CPT codes 33517-33548) from 2013 to 2022. CABG, coronary artery bypass graft Figure Credits: Jack Allen and Alexander Dorius

Each procedural group accounted for varying proportions of the total gross revenue for all cardiothoracic surgery CPT codes. Pacemaker and defibrillator procedures comprised 21.56%, valvular procedures comprised 26.49%, and CABG procedures comprised 41.24% of total gross revenue. Pacemaker and defibrillator (mean: -20.85%; median: -20.59%), valvular (-22.07%; -22.07%), and CABG (-21.99%; -22.06%) procedural groups had varying mean and median total national percent changes. Post hoc analysis revealed a significant difference between mean differences for the total national percent change in Medicare reimbursement across pacemaker and defibrillator, valvular, and CABG procedures. Valvular (mean difference: -1.21%) and CABG (mean difference: -1.14%) procedures decreased more compared to pacemaker and defibrillator procedures (p=0.01 and p=0.02, respectively), which is indicated in Figures 3, 4. There was no significant difference in Medicare reimbursement changes between valvular and CABG procedures.

There have been drastic changes in inflation-adjusted reimbursement for the analyzed procedural groups, especially for the years 2020, 2021, and 2022. Interestingly, there was a slight increase in average reimbursement rates for all procedural groups from 2013 to 2014. However, most of these changes follow the effects of inflation over the period while looking at the consumer price index. Overall, each procedural group observed a similar decline in reimbursement likely due to reimbursement rates not being adjusted for inflation.

## Discussion

This analysis indicates that inflation-adjusted Medicare reimbursement decreased by 2.67% annually between 2013 and 2022. Due to the increasing proportion of the United States population that is insured by Medicare, this data presents a concerning trend for cardiothoracic surgeons. Nearly 18.1% of the United States population is currently covered by Medicare, and that number is expected to increase due to the aging population. Though the exact proportion of patients covered by Medicare for all cardiothoracic procedures remains unclear, the changes in Medicare reimbursement have a substantial impact on private insurance reimbursement [13,14]. The results of this study provide context for healthcare policy lobbying positions that advocate for a sustainable future for cardiothoracic surgeons.

Geographic variance in Medicare reimbursement rates provides a compelling incentive for physicians to practice in higher reimbursement areas. This trend could lead to decreased access to care in low-reimbursement states such as Illinois or Kansas. Reimbursement rates may exacerbate existing healthcare disparities and, therefore, healthcare policy lobbying is necessary to ensure adequate reimbursement for all cardiothoracic surgery procedures [15,16]. Studies have found that lower reimbursement states with higher Medicaid enrollment report physicians with higher patient volumes [16]. Therefore, diminishing reimbursement rates may also prompt surgeons to leave certain localities, limiting access to cardiothoracic surgeons in specific states. Additionally, a future analysis should be conducted to examine the existence of a possible cut-off point in reimbursement after which physicians choose to leave certain geographic areas. Further study is needed to examine the impact of lower reimbursement on geographical variance in surgical outcomes in cardiothoracic surgery. Further research is also required to examine the effect of geographic variation in reimbursement on the decision of practice location.

Other studies have indicated that RVUs and payment do not always reflect the time and expertise required for certain procedures [14]. Due to lower physician payment, certain procedures are not performed as often and may limit access to care for some Medicare patients [17]. For example, in cardiothoracic surgery, aortic valve replacements are reimbursed at a higher level than aortic valve repair surgeries. Changes in the Medicare budget are often made by policy makers, few of whom understand the specifics of medical procedures. Awareness of reimbursement trends is an essential component to ensuring quality care for all Medicare patients. This analysis provides a data point for future lobbying to ensure adequate Medicare reimbursement for cardiothoracic surgery procedures. 

The primary limitation of this analysis is that the publicly available data on insurance reimbursement does not consist of third-party payors. Additionally, the analyzed codes account for almost all Medicare reimbursement for cardiothoracic surgery procedures, but there remains an insignificant contribution of low-grossing codes not considered in this analysis. The Medicare data reveals trends in overall reimbursement but does not consider private insurance companies that do not publish reimbursement data for open access. Future lobbying could also focus on increasing availability of private insurance data in order to more fully understand trends in reimbursement. Additional studies are required to evaluate how reductions in Medicare reimbursement influence private insurance payment structures. Further research should also explore the effects of geographic disparities in reimbursement on cardiothoracic surgeons, patient care, and hospital operations.

## Conclusions

Medicare reimbursement for cardiothoracic surgery procedures decreased from 2013 to 2022. Our study confirms that reimbursement patterns vary by geographical area, which may present a financial incentive for cardiothoracic surgeons to practice in specific areas. There were significant differences in Medicare reimbursement changes between several high-grossing cardiothoracic procedural groups. The results of this study can provide context for healthcare policy decisions to ensure sustainable reimbursement for cardiothoracic surgeons across the United States. The findings suggest the need for increased awareness among policymakers and the development of new policies to encourage private insurers to share data. Such initiatives could involve applying pressure for transparency and engaging patient advocacy groups to lobby for policy changes that address the underlying issues.
